# Surgical Management of a Spontaneous Hepatocellular Carcinoma Rupture in a Non-cirrhotic Liver: A Case Report

**DOI:** 10.7759/cureus.83441

**Published:** 2025-05-04

**Authors:** Maria Isabel Manso, Henrique Morais, Ruben Martins, Mafalda Machado, Gildasio Martins Dos Santos

**Affiliations:** 1 General Surgery Department, Unidade Local de Saúde (ULS) Algarve, Faro, PRT; 2 General Surgery Department, Hospital de Loulé, Loulé, PRT; 3 Radiology Department, Centro Hospitalar Universitário do Algarve, Faro, PRT

**Keywords:** acute care surgery, hemoperitoneum, hepatocellular carcinoma (hcc), liver resection, non-cirrhotic liver

## Abstract

Spontaneous rupture of hepatocellular carcinoma (HCC) is an uncommon and life-threatening condition that poses significant diagnostic and therapeutic challenges. This report describes the case of a 78-year-old man with no prior history of liver disease who presented with acute abdominal pain and hemoperitoneum. Imaging revealed a ruptured exophytic HCC lesion in hepatic segment II, which was confirmed during laparotomy. The patient underwent successful segment II resection following stabilization, and histopathological analysis confirmed a moderately differentiated HCC in a non-cirrhotic liver. The postoperative course was uneventful. This case highlights the importance of timely diagnosis, tailored management strategies, and multidisciplinary care in addressing the complexities of spontaneous HCC rupture, particularly in patients without underlying liver disease.

## Introduction

Hepatocellular carcinoma (HCC) is a primary liver tumor and one of the more common malignancies worldwide, with an overall poor prognosis [1,2]. It most frequently develops in patients with cirrhosis, with viral infections and alcohol consumption being the leading risk factors [3]. However, approximately 20% of HCC cases occur in non-cirrhotic livers. These cases may be misdiagnosed and are often detected at an advanced stage [4,5].

The clinical presentation of HCC varies widely. In most instances, HCC is diagnosed during hospitalization for decompensated liver cirrhosis [1]. Acute rupture of HCC is a rare presentation, particularly in Western countries, where it accounts for less than 3% of cases. It occurs more commonly in patients with cirrhotic livers [1-9]. Spontaneous rupture of hepatocellular carcinoma is a rare but severe complication that can lead to life-threatening internal bleeding, particularly challenging to diagnose when the liver shows no signs of cirrhosis. According to the literature, HCC rupture is the third leading cause of death in affected patients, following tumor progression and hepatic failure. Acute-phase mortality rate is high, with 30-day mortality ranging from 32% to 75% [4,5,10-12].

The mechanisms underlying spontaneous HCC rupture are not fully understood, but several hypotheses have been proposed [13,14]. The normal hepatic parenchyma surrounding the HCC is believed to provide structural protection, making rupture less common in non-cirrhotic livers [8,15]. In most reported cases of ruptured HCC, chronic viral cirrhosis - particularly due to hepatitis B virus infection - was the predominant underlying etiology [15]. Additionally, subcapsular HCC lesions are more prone to rupture, even at smaller sizes, compared to centrally located tumors.

Spontaneous HCC rupture typically occurs abruptly, with patients presenting to emergency departments with acute abdominal pain or hemodynamic instability. This condition poses a diagnostic challenge for emergency physicians, and delays in diagnosis can significantly worsen prognosis [1,5].

This report describes a unique case of spontaneous HCC rupture in a patient with no prior history of liver disease, successfully managed through surgical resection of hepatic segment II.

## Case presentation

A 78-year-old man presented to the emergency department with sudden-onset abdominal pain, followed by syncope, which resulted in a mandibular fracture. His medical history was notable for a gastric ulcer and chronic alcohol consumption of 75 cc/day. On admission, the patient exhibited mild mental confusion, a blood pressure of 100/50 mmHg, a heart rate of 100 bpm, and preserved diuresis. Physical examination revealed a distended abdomen with tenderness in the epigastric region. Initial blood tests showed a hemoglobin level of 14.8 g/dL (reference range: 14 to 18 g/dL).

Twelve hours later, the patient’s abdominal tenderness persisted, and repeat blood tests revealed a hemoglobin level of 8.5 g/dL. Additional laboratory findings included thrombocytopenia (87,000/µL; reference range: 150,000 to 450,000/µL) and hyperbilirubinemia (1.9 mg/dL; reference range: 0.1 to 1.2 mg/dL), with no other significant abnormalities in liver function. Serologic tests for hepatitis B and C were negative, and there was no clinical or laboratory evidence suggestive of chronic liver disease.

**Figure 1:**
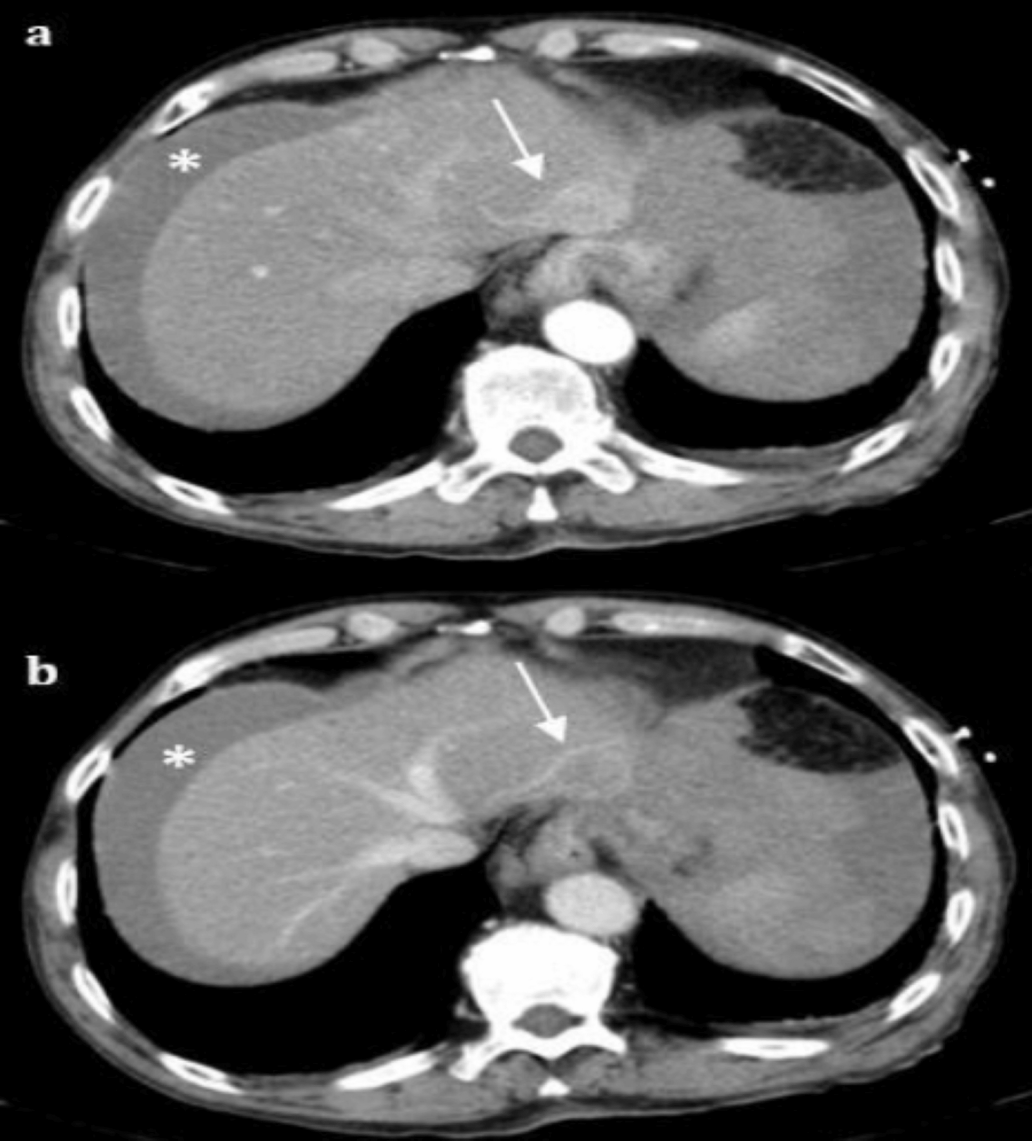
CT findings Contrast-enhanced computed tomography images showing the exophytic hepatic lesion in segment II. (a) The arterial phase demonstrates vivid enhancement of the lesion (arrow), while the portal venous phase (b) shows washout, both characteristic of hepatocellular carcinoma. Hemoperitoneum is visible and marked with an asterisk (*), indicating active intra-abdominal bleeding. These findings are consistent with spontaneous tumor rupture.

Contrast-enhanced computed tomography (CT) of the abdomen was performed and revealed a large hemoperitoneum and an exophytic liver lesion in segment II measuring 22 mm. The lesion demonstrated vivid enhancement during the arterial phase and washout during the portal venous phase, findings highly suggestive of HCC. The lesion had indistinct external borders and was identified as the probable source of bleeding (Figure 1)

**Figure 2:**
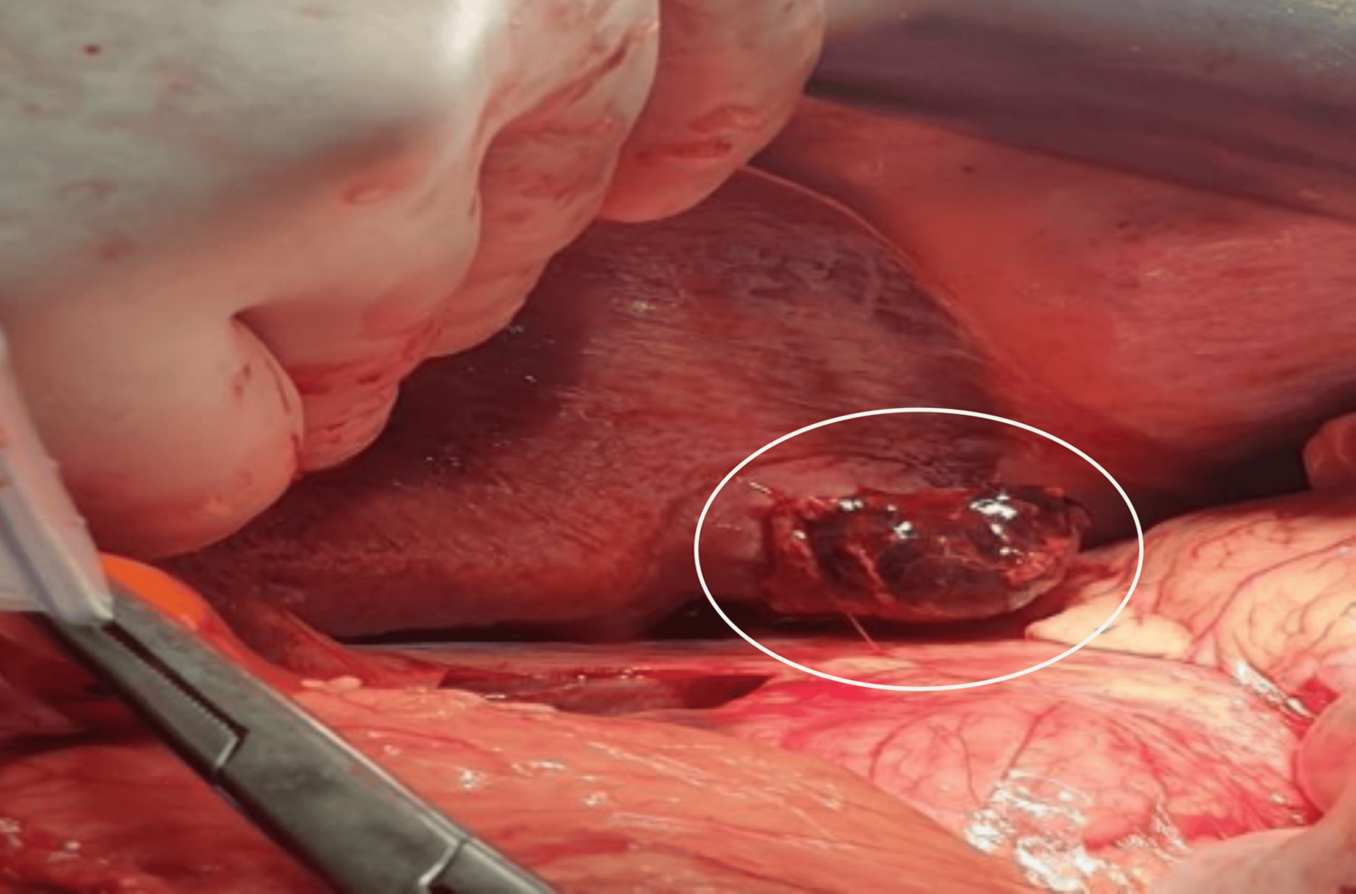
Intraoperative findings Intraoperative image of the hepatic segment II lesion, identified as the bleeding source. The lesion appears exophytic and ruptured at its inferior margin, correlating with imaging findings and clinical presentation.

**Figure 3:**
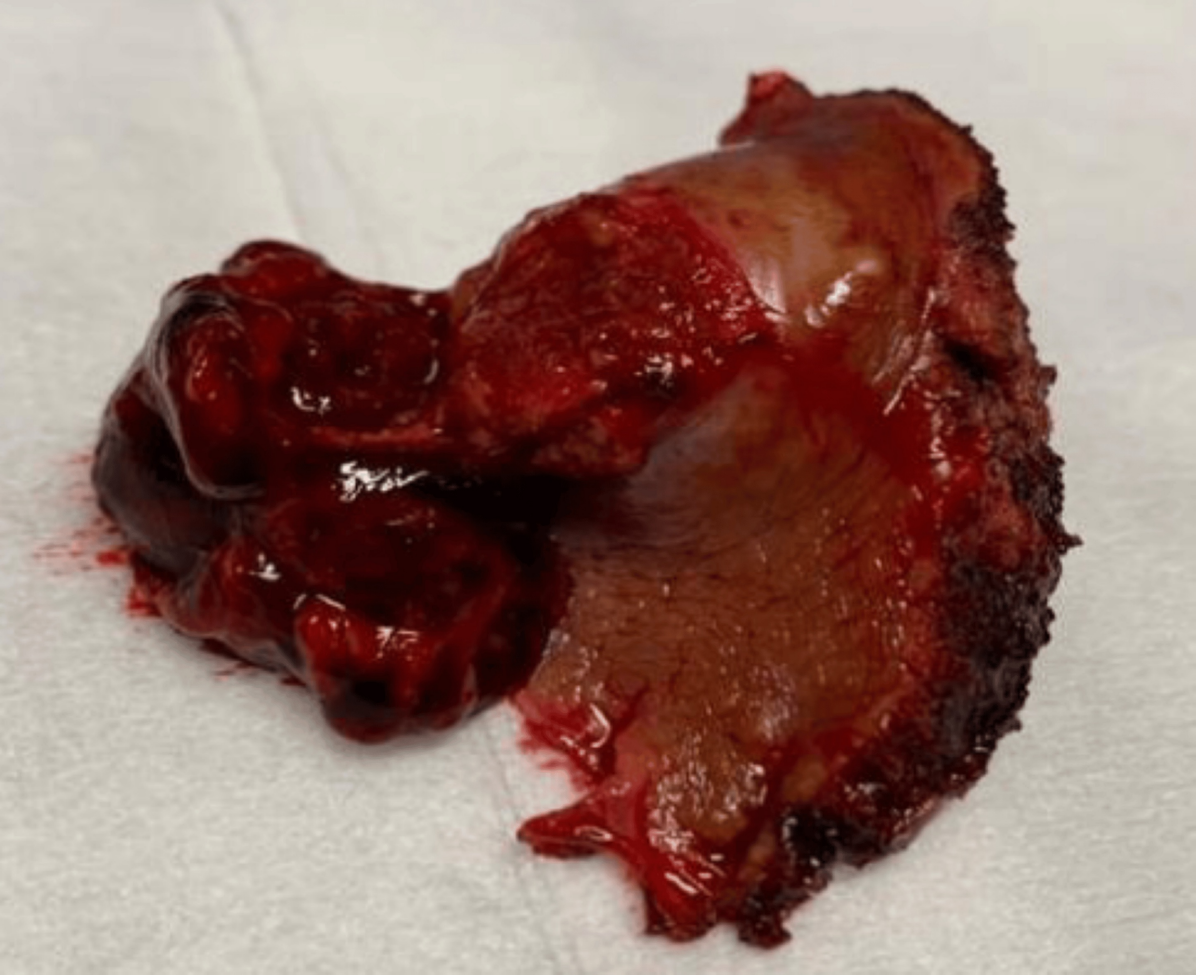
Image of segmentectomy II surgical specimen Surgical specimen from hepatic segment II resection. The exophytic tumor, measuring approximately 3 cm, shows surface disruption consistent with rupture. Histological analysis confirmed moderately differentiated hepatocellular carcinoma with clear surgical margins.

Given the lack of access to interventional radiology, the patient underwent exploratory laparotomy. The surgery revealed approximately 1500 cc of hemoperitoneum and an exophytic, ruptured liver tumor measuring 3 cm in diameter with active bleeding. The tumor was located on the inferior border of hepatic segment II, and the liver was non-cirrhotic. A Pringle’s maneuver was performed, followed by resection of segment II. Hemostasis was achieved using electrocautery, and a subhepatic drain was placed (Figures 2, 3).

Abdominal CT and intraoperative findings revealed a non-cirrhotic liver without nodular transformation or fibrosis, consistent with the absence of chronic liver disease despite a history of alcohol use.

The postoperative course was uneventful, and no blood transfusions were required. On postoperative day 4, the patient was transferred to the facial surgery department for management of the mandibular fracture. Follow-up evaluations revealed no complications. Histopathological analysis of the resected specimen confirmed a moderately differentiated HCC (pT4) with clear surgical margins.

## Discussion

Ruptured HCC is uncommon in Western countries, with an incidence of less than 3%, compared to 12% in Eastern countries. The reported mortality rate can reach up to 50% [14]. Several factors may increase the risk of spontaneous HCC rupture, including inappropriate use of antiplatelet or anticoagulant therapy, coagulopathy and thrombocytopenia associated with cirrhosis, extrahepatic invasion, tumors larger than 5 cm, rapid tumor growth, and advanced age [7]. While most cases of hepatocellular carcinoma arise in cirrhotic livers, chronic alcohol consumption has been identified as an independent risk factor for hepatocarcinogenesis, even in the absence of cirrhosis. In this case, the patient’s history of alcohol use likely contributed to carcinogenesis despite a non-cirrhotic liver architecture. Although these risk factors are well-documented, the occurrence of HCC rupture remains rare, highlighting the need for careful evaluation and management of at-risk patients.

Diagnosing ruptured HCC relies on a combination of clinical symptoms, physical findings, and imaging studies. Patients often present with signs of acute blood loss or hemorrhagic shock with abdominal tenderness, distension, or pain [1]. However, preoperative diagnosis can be challenging, particularly in individuals without a prior history of cirrhosis or HCC [7,14].

Abdominal ultrasonography and CT scans are critical in confirming the presence of hemoperitoneum and identifying liver tumors. Ultrasound is particularly useful in hemodynamically unstable patients, as it can confirm hemoperitoneum and guide subsequent imaging. CT provides a more detailed assessment of tumor characteristics and bleeding, while angiography can reveal active extravasation of contrast - a hallmark of HCC rupture - although this finding is only observed in 13.2% to 35.7% of cases [4,7,13,15,16]. Indirect signs, such as subcapsular hematoma, hemoperitoneum, ascites, or tumor contour defects, further support the diagnosis of ruptured HCC [1,17,18].

Managing ruptured HCC remains a complex and controversial issue, with no established consensus on the optimal approach. Initial treatment focuses on resuscitation of hypovolemic shock and bleeding control while preserving liver function [2,4,9,14,19,20]. The selection of definitive treatment depends on multiple factors, including hemodynamic stability, liver function, tumor characteristics, and disease stage [3,8]. Hemodynamically stable patients without active bleeding are typically managed conservatively, followed by definitive treatment such as liver resection or transarterial chemoembolization. In contrast, hemodynamically unstable patients require immediate intervention to control bleeding.

Conservative management necessitates a thorough assessment of liver function and tumor stage to determine eligibility for transarterial embolization (TAE) or hepatic resection. While TAE is minimally invasive and highly effective, it carries a risk of continued or recurrent bleeding [5,7,15]. TAE is particularly beneficial for patients with advanced liver disease (Child-Pugh class C), multifocal or bilobar HCC, or tumors located in surgically challenging positions. It may also serve as a bridging therapy, stabilizing patients until they are eligible for surgical resection [1,2,4,13]. Despite its benefits, TAE is not without complications, which include rebleeding, abscess formation, and tumor recurrence

Surgical resection remains the optimal treatment for selected patients with good performance status, stable hemodynamics, and preserved liver function. Studies have shown that emergency liver resection is effective in patients with relatively preserved liver function, particularly for small, superficial, and accessible tumors in non-cirrhotic livers [4,5,14,15]. Other interventions, such as perihepatic packing, suture plication of the bleeding tumor, alcohol injection, and hepatic artery ligation, may be employed in cases of extensive hepatic disease, hemodynamic instability, or tumors unsuitable for resection [13,15].

**Figure 4:**
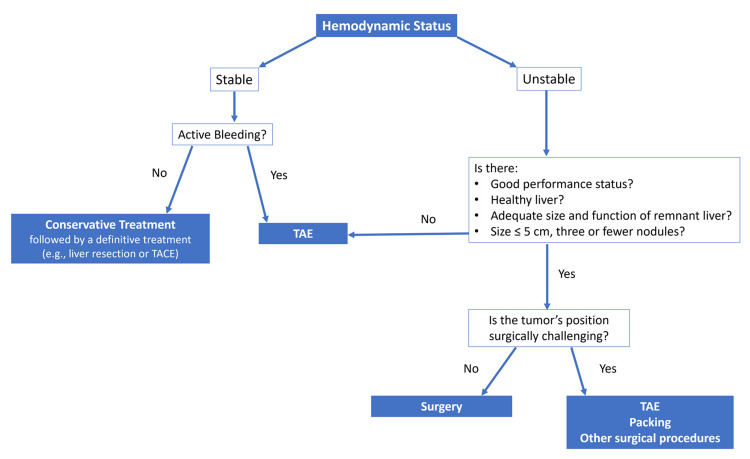
Management algorithm for spontaneous rupture of hepatocellular carcinoma based on hemodynamic stability and tumor characteristics Management algorithm outlining treatment strategies for spontaneous HCC rupture, based on hemodynamic stability, tumor location, liver function, and available resources. The chart provides a simplified decision-making pathway, highlighting when to consider conservative management, transarterial embolization (TAE), or surgical resection Abbreviations: TAE, transarterial embolization; TACE, transarterial chemoembolization; HCC: hepatocellular carcinoma. Image credits: Maria Isabel Manso

In the present case, the patient underwent segment II liver resection due to stable hemodynamics, good performance status, a healthy liver, and a small lesion (<5 cm) in a surgically favorable location. Based on the staging of liver cancer, the tumor was classified as stage A, which is acceptable for liver resection. This case highlights the importance of individualized treatment strategies, considering hemodynamic stability, liver function, and tumor characteristics. A comprehensive approach to diagnosis and management is crucial to optimizing outcomes in patients with ruptured HCC (Figure 4).

## Conclusions

We present the case of a 78-year-old man who presented with acute abdominal pain and hemoperitoneum. Laparotomy revealed blood in the abdominal cavity originating from a ruptured tumor in hepatic segment II, consistent with spontaneous HCC rupture. Segmentectomy II was successfully performed, and the patient experienced an uneventful postoperative course. The diagnosis of spontaneous HCC rupture remains challenging, particularly in patients without cirrhosis. Rapid diagnosis is essential and often facilitated by imaging studies and laboratory analyses; however, delays in diagnosis still occur, especially in non-cirrhotic cases. Treatment strategies should focus on stabilizing the patient, controlling bleeding, and preserving liver function, with emergency liver resection being an effective option for selected patients. This case highlights the importance of a multidisciplinary approach in managing ruptured HCC to achieve favorable outcomes in this rare but life-threatening condition.
